# MDCT of acute subaxial cervical spine trauma: a mechanism-based approach

**DOI:** 10.1007/s13244-014-0311-y

**Published:** 2014-02-21

**Authors:** Sameer B. Raniga, Venugopal Menon, Khamis S. Al Muzahmi, Sajid Butt

**Affiliations:** 1 Division of Radiology, Khoula Hospital, PO BOX 794, Muscat, 117 Oman; 2 Orthopedic and Spine Surgery, Khoula Hospital, PO BOX 794, Muscat, 117 Oman; 3 Division of Radiology, Royal National Orthopaedic Hospital, Stanmore, London, UK

**Keywords:** Biomechanics, Multidetector computed tomography, Cervical vertebrae, Cervical spine injury, Spinal cord injury

## Abstract

Injuries to the spinal column are common and road traffic accidents are the commonest cause. Subaxial cervical spine (C3–C7) trauma encompasses a wide spectrum of osseous and ligamentous injuries, in addition to being frequently associated with neurological injury. Multidetector computed tomography (MDCT) is routinely performed to evaluate acute cervical spine trauma, very often as first-line imaging. MDCT provides an insight into the injury morphology, which in turn reflects the mechanics of injury. This article will review the fundamental biomechanical forces underlying the common subaxial spine injuries and resultant injury patterns or “fingerprints” on MDCT. This systematic and focused analysis enables a more accurate and rapid interpretation of cervical spine CT examinations. Mechanical considerations are important in most clinical and surgical decisions to adequately realign the spine, to prevent neurological deterioration and to facilitate appropriate stabilisation. This review will emphasise the variables on CT that affect the surgical management, as well as imaging “pearls” in differentiating “look-alike” lesions with different surgical implications. It will also enable the radiologist in writing clinically relevant CT reports of cervical spine trauma.

*Teaching Points*

• *Vertebral bodies and disc bear the axial compression forces, while the ligaments bear the distraction forces*.

• *Compressive forces result in fracture and distractive forces result in ligamentous disruption*.

• *Bilateral facet dislocation is the most severe injury of the flexion-distraction spectrum*.

• *Biomechanics-based CT reading will help to rapidly and accurately identify the entire spectrum of injury*.

• *This approach also helps to differentiate look-alike injuries with different clinical implications*.

## Introduction

Injuries to the spinal column are frequently seen in clinical practice. The commonest cause of these injuries is road traffic accidents [1]. Falls, assault, penetrating and sports injuries form the remaining causes of spinal injury. The prevalence is likely to be significantly higher in patients with head trauma and those who are unconscious at presentation [2, 3]. The subaxial cervical spine (C3–C7) is particularly vulnerable to traumatic injury due to its considerable mobility and its close proximity to the more rigid thoracic spine. This region accounts for about 65 % of fractures and more than 75 % of all dislocations in the spine, with fractures occurring most often at C6 and C7, and dislocations occurring most frequently between C5–C6 and C6–C7 vertebrae [1–3]. Injuries of the subaxial cervical spine occur along a wide spectrum of severity, from minor soft tissue “strains” to disastrous fracture dislocations with extensive spinal cord mutilation.

MDCT has gained widespread acceptance in the evaluation of spine trauma and many centres use MDCT as the initial and definite imaging modality in cervical spine trauma [4]. MDCT offers volume imaging with isotropic reconstruction, and provides quick and efficient imaging. In a polytrauma case, a single data acquisition can generate a spine dataset, visceral dataset and limb trauma data as indicated [5–7].

At our institution, cervical spine CT in trauma is performed with a 64-detector CT scanner with a 0.625-mm detector configuration from clivus to T3. The isotropic dataset is used to generate multiplanar reformats and 3D reconstruction. Two-millimetre axial, coronal and sagittal reformats are generated in both soft tissue and bone algorithms. CT angiography for assessment of the vertebral artery is performed when vertebral artery injury is suspected.

Cervical spine injuries occur in a specific and predictable pattern that is generally dependent on the mechanism of injury. The pattern may be easily recognised on imaging studies, referred to as the “fingerprints” of the injury— a concept popularised by Daffner et al. [8–11]. Considering the injury mechanism and understanding the patho-anatomy are important for the surgical decision-making process. Mechanism-based image interpretation of the cervical spine will use the easily detected lesions like fracture and dislocation to predict subtle but important abnormalities, which can potentially be missed.

In this article we review the fundamental traumatic forces that cause subaxial cervical spine injuries and illustrate their associated MDCT findings. Subsequently, we apply this knowledge to the analysis of cervical spine CT images and show how an understanding of the injury mechanisms can lead to the diagnosis of subtle but important abnormalities.

## Structural and functional anatomy of subaxial cervical spine

**Fig. 1:**
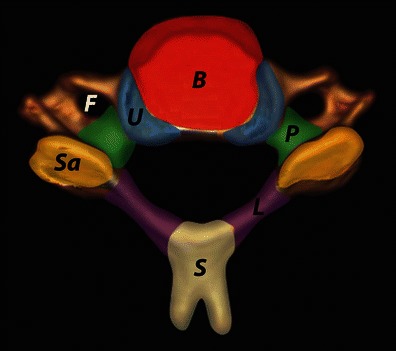
Typical cervical vertebra anatomy. Vertebral body (*B*) is anteriorly located (*red*)—cylindrical in shape, pedicles (*P*) are directed posterolaterally (*green*), laminae (*L*) are directed posteromedially (*purple*) and give rise to spinous process (*S*) with bifid tip (*light yellow*). Vertebral canal is triangular. Transverse processes contain vertebral foramen (*F*) and vertebral artery passes through it. Lateral masses are seen at the junction of pedicle and lamina—contains the articular facet at the superior (*Sa*, *orange*) and inferior aspect. Uncinate process (*U*) arises from the posterolateral corner of the vertebral body’s superior surface (*blue*)

**Fig. 2:**
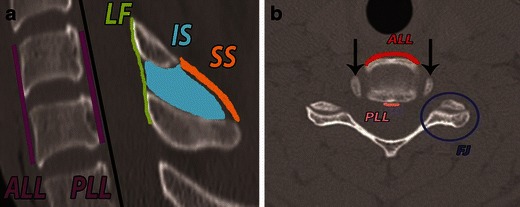
Colour-coded schematic shows disco-ligamentous complex and spinal motion segment. **a** Spinal motion segment consists of two adjacent vertebrae connected together by the joints and ligaments. Ligamentous restrainers from anterior to posterior include: anterior longitudinal ligament (*ALL*), intervertebral disc, posterior longitudinal ligament (*PLL*), ligamentum flava (*LF*), interspinous (*IS*) and supraspinous (*SS*) ligaments. Intervertebral disc, facet and uncovertebral joints stabilise the motion segment. **b** Axial CT image shows the stabilising ligaments, ALL and PLL, the facet and the uncovertebral joints. Uncinate processes (*black arrows*) are in symmetrical, concentric relationship at the posterolateral aspect of the cranial vertebra. The facet joint on axial image resembles a “hamburger bun” with the flat surface articulating

**Fig. 3:**
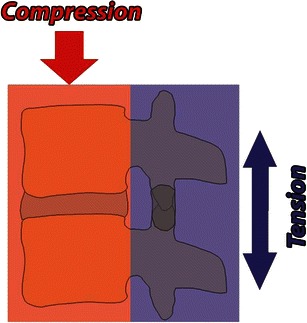
The “two vertical column” concept of spine biomechanics and stability. In the two-column concept, the anterior column constitutes the ALL, vertebral body, intervertebral disc and PLL. The posterior column constitutes bony neural arch and posterior ligamentous complex (*PLC*). Load bearing anterior column resists compression forces. The posterior ligamentous complex including facet joints forms the principle tension band and resists distraction forces

The subaxial cervical spine includes the C3–C7 vertebrae. A typical cervical vertebra has an anterior vertebral body and a posterior neural arch (Fig. 1). The discoligamentous complex (DLC) consists of intervertebral discs and spinal ligaments that bind adjacent vertebrae to each other and guide segmental motion and contribute to the intrinsic stability of the spine by limiting excessive motion. Anatomical components of the DLC include the anterior longitudinal ligament (ALL), intervertebral disc, posterior longitudinal ligament (PLL), facet joint capsule, ligamentum flava, interspinous and supraspinous ligaments (Fig. 2). A functional spinal unit (FSU) or spinal motion segment is the smallest anatomical unit of the spine to exhibit the biomechanical characteristics of the entire spine [12, 13]. The spine motion segment consists of two adjacent vertebrae connected together by the DLC. Biomechanically, the spinal motion segment can be divided into anterior and posterior columns [12, 14]. The anterior column is anterior to PLL and includes the vertebral body, intervertebral disc, ALL and PLL. Posterior column is posterior to the PLL and consists of the neural arch and the posterior ligamentous complex (PLC) (Fig. 2). The PLC is the primary tension band of the spinal motion segment preventing hyperflexion, torsion and anterior–posterior shear or translation [15, 16]. The ALL is the strongest anterior ligamentous structure preventing hyperextension [17, 18]. Vertebral bodies and intervertebral discs bear the compressive loads on the spine, while the ligaments bear rotational, distractive and shear forces (Fig. 3) [14, 15].

## Biomechanics: basic concepts for radiologists

Most of the severe cervical spine injuries result from direct contact from head impact. Non-contact injuries to the neck are commonly referred to as whiplash injuries and result from unrestrained neck motion during a motor vehicle collision. Within a given injury mechanism, there is a spectrum of injury, a phylogeny, which ranges from trivial to severe [19]. Mechanical subclasses are possible depending on the instant centre of rotation (fulcrum) at the moment of impact. Similar forces will result in different types of injury depending upon the fulcrum, which in turn is determined by the position of the head and neck at the time of impact.

**Fig. 4:**
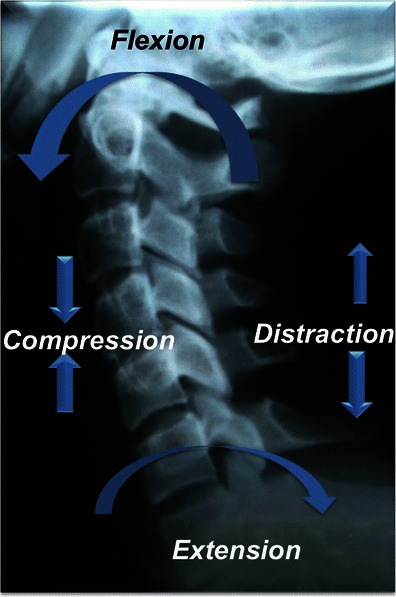
Schematic shows major injury vectors and forces with their impacts on the vertebral column. Flexion injury results from supraphysiological forward bending and extension injury results from backward banding. Compression forces approximate the bones, while distraction dissociates the bones. Shear forces are applied at a right angle to the long axis of spine and produces significant bony and ligamentous disruption

In general, compressive forces result in burst or wedge fractures; distractive forces result in ligamentous disruption or dislocations, while rotation-shear forces result in combined bony and ligamentous injuries producing fracture-dislocation (Fig. 4).

## Systematic review of MDCT of cervical spine in trauma—“checklist”

Missing a cervical spine injury can have potentially catastrophic neurological consequences or mechanical instability. To avoid search pattern errors on MDCT, it is helpful to have a checklist approach that will ensure that all the important structures are scrutinised for normality, and abnormality is detected if present and further characterised (Table 1).

**Table 1:** Cervical spine trauma CT interpretation: checklist

	Anterior column	Posterior column
Bone	Vertebral body:	Neural arch:
	Normal—preserved height without wedging, smooth cortices without step Abnormal—fracture Further description—comminution, Involvement of posterior cortex of vertebral body, retropulsion of fragments	Normal—intact without fracture Abnormal—fracture Further description: comminution, Orientation of fracture line—vertical vs horizontal; unilateral vs bilateral; symmetrical vs asymmetrical; isolated posterior elements or associated with anterior column fractures
Joints	Intervertebral disc:	Facet joint:
	Normal—symmetrical disc space without focal widening or narrowing Abnormal—focal anterior or posterior widening or asymmetry in coronal plane	Normal—congruent, parallel and symmetrical Abnormal—diastasis, subluxation, dislocation; focal anterior vs posterior widening Further description—unilateral vs bilateral Associated articular process fracture
Ligaments	ALL/PLL:	PLC:
	Normal integrity inferred from alignment of anterior posterior vertebral lines Abnormal—anterior vs posterior translation. Triangular avulsion at the corners of vertebral end plates	Normal- Integrity inferred from the normal alignment, interspinous distance and facet joint morphology Abnormal—Anterolisthesis, focal kyphosis, interspinous widening and facet joint abnormality as mention in the previous column
Curvature	Normal—smooth lordosis Abnormal—focal kyphosis	–
Alignment	Anterior/posterior vertebral body lines in sagittal plane	Spinolaminar and interspinous lines on sagittal plane Interspinous and articular pillar lines in coronal plane
Measurement	–	Interpedicular distance. Interspinous and interlaminar distance Abnormal: difference of more than 2 mm

**Fig. 5:**
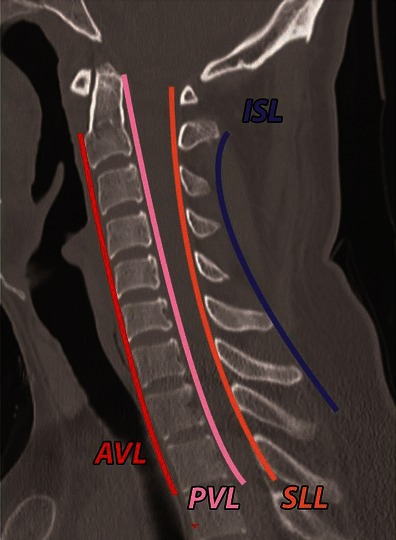
Normal alignment of the spine as seen on mid-sagittal CT: anterior vertebral line (*AVL*, *red*)—connecting the anterior cortices of the vertebrae; posterior vertebral line (*PVL*, *pink*)—connecting the posterior cortices of the vertebrae; spinolaminar line (*SLL*, *orange*)—connecting the base of the spinous processes at the spinolaminar junction; interspinous line (*ISS*, *blue*)—connecting the tips of the spinous processes. All of these lines should be gently curved, smooth and continuous

The interpreting physician must be aware of the appropriate CT imaging plane for the optimal visualisation of different components of the checklist and further characterisation of abnormality (Table 2). In the sagittal plane, bony alignment on CT is evaluated by drawing: the anterior vertebral line—connecting the anterior vertebral cortices; the posterior vertebral line—connecting the posterior vertebral cortices; the spinolaminar line—a smooth curve from opisthion to C7 formed by junction of laminae with spinous processes; the interspinous line—connecting the tip of the spinous processes of C3–C7. All of these lines should be in a smooth curve without focal discontinuity or angulation (Fig. 5). On the sagittal plane, the posterior vertebral line is the most reliable and accurate indicator of antero-posterior alignment.

**Table 2:** CT checklist and optimal plane of visualisation

Fingerprint	Sagittal	Axial	Coronal
Alignment	Anterior and posterior translation Anterior and posterior vertebral line, spinolaminar line, interspinous line	Uncinate process alignment—for assessment of rotation deformity	Lateral translation of vertebral body Articular pillar alignment Spinous process alignment for rotation
Bone (fractures)	Vertebral body compression—wedging Coronal split fracture Retropulsion Articular pillar and articular process fracture Spinous process fracture	Comminution of vertebral body Sagittal/coronal split fractures Retropulsion Pedicle and lamina fracture. Transverse process fracture	Sagittal split fracture Lateral cortical step of vertebral body Articular pillar Transverse process Any horizontally oriented fracture
Measurement	Interspinous widening Interlaminar widening Kyphotic angle	–	Interpedicular widening Interspinous widening (sagittal is preferred)
Joint	Intervertebral disc Facet joints	Uncinate process alignment for rotation Facet joint (sagittal is preferred)	Intervertebral disc asymmetry in coronal plane—suggestive of rotation and lateral flexion vector
Ligaments	ALL, PLL, PLC injury—fingerprints	–	–

Volume rendered 3D images are useful in detecting- transverse process, spinous process fracture and detection of rotation of the vertebral body

**Fig. 6:**
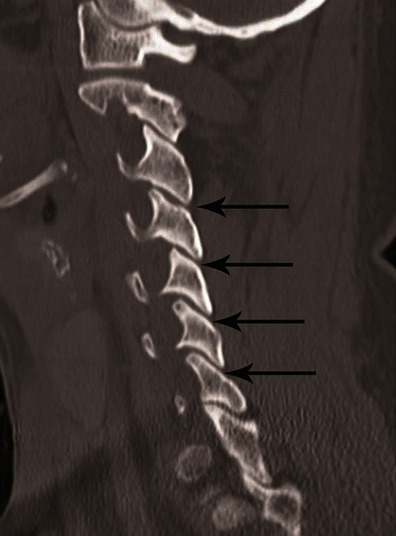
Normal alignment of facet joints on parasagittal CT image (*arrows*). A facet joint is formed by the obliquely oriented rhomboid-shaped articular processes and overlaps like a roof shingle. The inferior articular process of the cranial vertebra is posterior to the superior articular process of the caudal vertebra. On sagittal images, a facet joint articular surface should be parallel, cranio-caudally symmetrical and congruent; the inferior articular facet should cover the entire articular surface on the superior articular facet below. The facet joint gap should be less than 2 mm

Facet joints are best evaluated in the sagittal plane on parasagittal images (Fig. 6). On axial CT images, the facet joints resemble a hamburger bun, with the flat portions articulating (Fig. 2) [20]. The uncinate processes of the lower vertebra lie lateral to the cranial vertebral body (Fig. 2) [21].

In the coronal plane, the lateral masses make bilateral smooth undulating margins. Spinous processes are in midline and any focal displacement of one spinous process in relation to the others is abnormal. Each of the bony components of the cervical vertebrae should be evaluated for fractures on all the available images in different planes and 3D reconstruction.

## CT measurements

Quantification of the angulation, deformity, dislocations and measurement of prevertebral soft tissue to detect the abnormality or instability has become less relevant in modern practice, as MDCT shows the entire spectrum of the osseous and ligamentous disruption in multiple planes, which previously had to be inferred indirectly from the radiographs previously. Moreover, these measurements have been shown to be inconsistent with limited interobserver reliability [22]. However, they can be measured on MDCT, which can be included in the checklist. To simplify and memorise different measurement parameters, Daffner and Harris [23] have popularised the “Rule of 2s”. Interspinous, interlaminar and interpedicular distances, and also facet joint width, are abnormal if the difference of these parameters between the adjacent segments is more than 2 mm. Interlaminar distance is more reliable and accurate than interspinous distance for diagnosing hyperflexion injury.

## Common injury patterns

In this section the spectrum and pattern of injury produced by different forces on the cervical spine and their typical findings on CT scan is described.

### Hyperflexion injury

Hyperflexion is the most frequent type of injury vector encountered in patients with vertebral trauma. The injury spectrum includes compressive hyperflexion, vertical compression and distractive hyperflexion. The injury pattern resulting from these force vectors depends upon the centre of rotation, the fulcrum, which in turn is determined by the position of the head and neck at the time of injury. Additional vectors like rotation and lateral flexion can result in complex hyperflexion-rotation injury.

#### Compressive hyperflexion

**Fig. 7:**
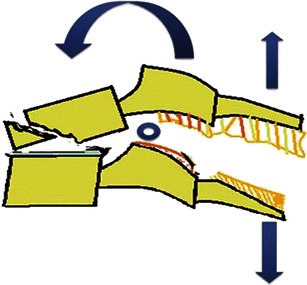
Schematic showing a hyperflexion compression injury. Flexion vector with centre of rotation (*blue hollow circle*) is just behind the anterior column, resulting in a compressive force applied to the anterosuperior aspect of the vertebral body, causing a primary progressive compressive failure of anterior column. With increasing magnitude of force, posterior column distraction and tensile failure result

**Fig. 8:**
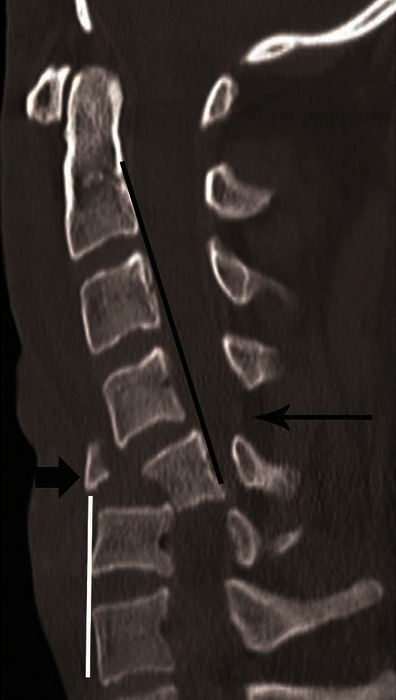
Flexion teardrop fracture. Sagittal CT image of cervical spine in a young patient following vehicular accident shows wedging of C5 vertebral body with an oblique coronal fracture of the anterior third of the vertebral body, dividing the C5 in to a smaller anterior triangular fragment (*short arrow*) and a large posterior segment. Retrolisthesis of the C5 vertebral body on C6 behind the fracture line and retropulsed C5 keeping alignment with the cranial C4 vertebral body—suggested by non-interrupted posterior vertebral line (*black line*), while triangular fragment keeping alignment with the caudal C6 vertebra—suggested by non-interrupted anterior spinal line (*white line*). Distraction of posterior column is suggested by widening of interspinous/interlaminar space at C4–C5 (*long arrow*). Facet joint subluxation was seen on the parasagittal images (not shown)

This represents axial loading in which the motion segment is flexed and a compressive force is applied at the anterosuperior margin of the vertebral body, the centre of rotation remaining in front of the anterior column, resulting in compressive failure of the anterior column (Fig. 7). These injuries begin with anterior vertebral body wedging. With increasing flexion compression force, anterior vertebral body develops a vertically oriented fracture line in the coronal plane, separating a triangular anterior “teardrop” fragment from the posterior vertebral body. The shape of this teardrop fragment is commonly triangular, but a quadrangular variant has also been described [24]. With increasing force, tensile failure of the posterior disc, PLL and PLC occurs resulting in a highly unstable injury. This is often described as a flexion teardrop fracture, the most severe form of hyperflexion injury with fracture-dislocation of the involved motion segment (Fig. 8). In general, this type of injury has the highest rate of neurological injury of all cervical injuries. Since flexion teardrop fracture results in global disruption of all the supporting ligaments, it is highly unstable and needs surgical stabilisation. CT characteristics of flexion teardrop fracture include [19, 25]: (1) oblique fracture in the coronal plane extending from anterior aspect of superior end plate to the inferior end plate—dividing the involved vertebral body into a small anterior triangular/quadrilateral fragment and a larger posterior body fragment; (2) posterior subluxation of the remaining posterior vertebral body on the lower vertebra—dividing the spine into a caudal segment consisting of the anterior teardrop fragment and the lower vertebral body and, a cranial segment consisting of the retropulsed fractured vertebral body with the vertebrae above; (3) signs of posterior ligament complex injury (Fig. 8).

#### Vertical compression/axial loading

**Fig. 9:**
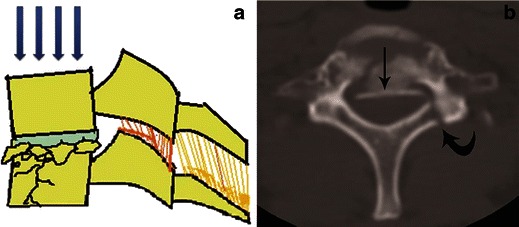
Axial loading (vertical compression injury). **a** Schematic showing a vertical compression injury. Compressive force is exerted vertically to the spinal column with neck in the neutral position (30-degree flexion) at the time of injury, resulting in pure axial loading injury and compressive failure of the anterior column. The posterior column remains intact or with increasing force fails in compression. **b** Axial CT shows marked comminution of the vertebral body with retropulsion of the fracture fragment from the posterior cortex in to the spinal canal (*straight arrow*) suggestive of burst fracture. Associated unilateral laminar fractures (*curved arrow*) are commonly seen

Axial loading is also referred to as vertical compression. This occurs when sufficient compressive force is exerted vertically through the spinal column (Fig. 9a). Injury from this mechanism often occurs with shallow diving, head-first tackling (rugby injury) or vehicular accidents with neck in neutral position (30 degrees of flexion). The vertebral bodies and intervertebral discs attempt to absorb the energy and literally burst, with horizontal spread of the fragments. Bone fragments are dispersed centrifugally in all directions, including into the spinal canal, often resulting in catastrophic neural damage. Pure axial loading forces lead to a symmetrical loss of vertebral body height, with little kyphosis or translation.

**Fig. 10:**
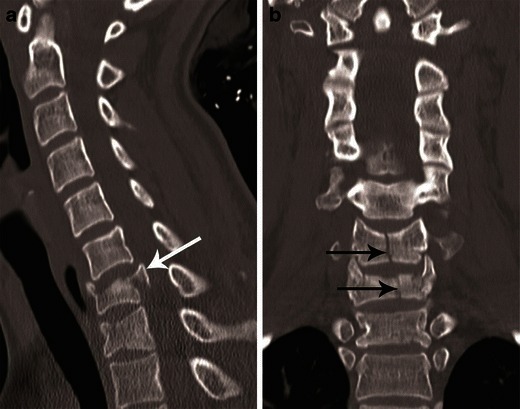
Burst fracture. **a** Mid-sagittal CT image showing a comminuted fracture of C7 with nearly symmetrical loss of vertebral body height with extension of the fracture to the posterior cortex and retropulsion of fragment from the posterospuerior cortex of C7 (*white arrow*)—suggestive of a burst fracture. Vertebral body comminution is better seen on axial images as shown in Fig. 9b. **b** Coronal CT image showing a vertical split fracture of C6 and C7 (*black arrows*). These fractures are oriented in the sagittal plane, hence best seen on coronal images and highly suggestive of axial loading injury

On CT, minor vertical compression results in central cupping of vertebral end plates and/or sagittal/coronal split fractures. With increasing force, a typical burst fracture shows vertebral body height loss with comminution of vertebral body and centrifugal displacement of the fracture fragments; fracture lines extending to the posterior cortex with retropulsed fragments (Figs. 9b and 10) [23].

#### Distractive hyperflexion

**Fig. 11:**
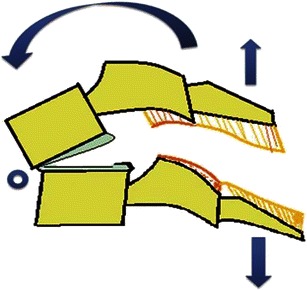
Schematic showing hyperflexion distraction injury. Flexion vector with centre of rotation (*blue hollow circle*) anterior to the anterior column, resulting in posterior column distraction with progressive tensile failure of the posterior column

The centre of rotation of this hyperflexion injury is thought to occur anterior to the vertebral body with neck in flexion, resulting in primary progressive tensile failure of posterior elements (Fig. 11) [19]. A minor compressive vector is also frequently present, resulting in fracture of the vertebral end plate. Flexion-distraction injuries represent a continuum of ligamentous injuries, ranging from sprain to complete tear of the PLC. Corresponding ligamentous disruption progresses from posterior to anterior, beginning with disruption of the supraspinous ligament, interspinous ligaments, facet capsular ligaments and ligamentum flava, in that sequence. With more severe distractive force, progressive failure of the anterior ligaments might also occur. Minor injuries of PLC can easily be missed and may result in delayed instability if overlooked on initial CT examinations.

**Fig. 12:**
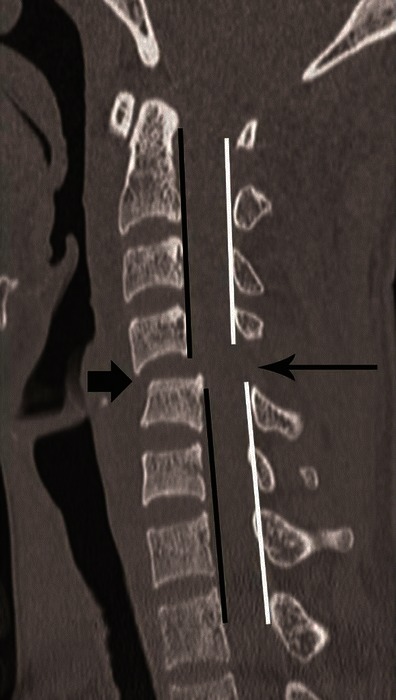
Hyperflexion distraction injury. Mid-sagittal CT image of the cervical spine shows focal kyphosis with posterior disc space widening at C4–C5 (*short arrow*). Mild anterolisthesis of C4 over C5 is seen suggested by the disrupted posterior vertebral line (*black lines*). The spinolaminar line is also disrupted with at C4–C5 with anterior displacement of C4 spinolaminar junction (*white lines*). Widening of the C4–C5 interlaminar/interspinous space (*long arrow*) is noted. Combination of this pattern of injury is highly suggestive of hyperflexion distraction injury and the facet joint should be carefully evaluated on lateral parasagittal images for possible distraction, subluxation and dislocation (shown in Fig. 13)

**Fig. 13:**
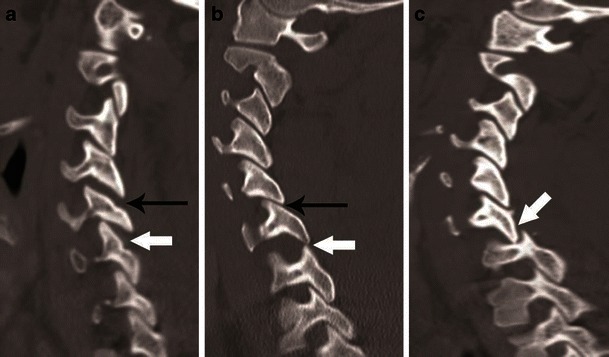
Facet joint injury spectrum resulting from hyperflexion distraction. Parasagittal CT images. **a** The C4–C5 facet joint shows diffuse widening—diastasis (*black arrow*), and the C5–C6 facet joint shows focal anterior widening (*white arrow*), suggestive of distraction injury and partial disruption of facet joint capsule. Articular surfaces are congruent and no uncovering of the inferior articular process noted at any of the injured levels. **b** C5–C6 facet joint subluxation (*black arrow*), suggested by non-congruent articular surfaces of the facet joint with anterosuperior displacement of the inferior articular process of C5, resulting in partial uncovering of the superior articular surface of C6. However, some apposition of articular surface is still intact. C6–C7 facet joint dislocation (*white arrow*) suggested by anterior and superior translation of inferior articular process of C6 resting on the top of the C7 articular process, resulting in complete loss of articular apposition and uncovering of C7 articular facet. A facet joint injury as seen at C6–C7 is also named as a “perched facet”. **c** C6–C7 facet joint dislocation with further anterior translation of the inferior articular process (*white arrow*), now resting anterior to the C7 articular pillar. This injury is also called a “locked” or “jumped facet”

CT features of PLC injury include: interspinous widening, interlaminar widening; facet joint distraction subluxation or dislocation, widened posterior disc space, focal kyphosis and anterior subluxation of the vertebral bodies (Figs. 12 and 13) [26]. The facet joint injury spectrum of hyperflexion distraction injury with progressive severity includes: facet joint distraction, subluxation and dislocation (Fig. 13). “Locked”, “perched” and “jumped” facet joints are alternative terminologies used in the radiology and surgery literature for the dislocated facet joint and should preferably be replaced by “facet joint dislocation”. Facet joint dislocation can be a purely ligamentous injury when predominant force is distraction, and this results in dislocation without fracture of the facets. In the presence of a shear or rotation vector, facet joint dislocation is accompanied by fracture of articular processes.

Bilateral facet dislocation is the most severe injury of the flexion-distraction spectrum, which usually results in profound neural insult [27]. Bilateral facet joint dislocation results in complete disruption of facet joint capsule and PLC in all cases. PLL disruption is also described in 40–100 % of cases of bilateral facet joint dislocation [27, 28]. Traumatic disc herniation with posterior annulus disruption is described in 56 % of unilateral and 82.5 % of bilateral facet dislocations [27]. Disc herniation is relevant in the management of these injuries and should be confirmed by magnetic resonance imaging (MRI) before the reduction or stabilisation of facet joint dislocation is attempted.

*Unilateral facet joint dislocation* is a hyperflexion injury when the fulcrum is off-centre. This results in simultaneous hyperflexion and axial rotation with asymmetric injury leading to asymmetrical tensile failure of posterior column and unilateral facet joint injury with fractures, subluxation and dislocation [29].

**Fig. 14:**
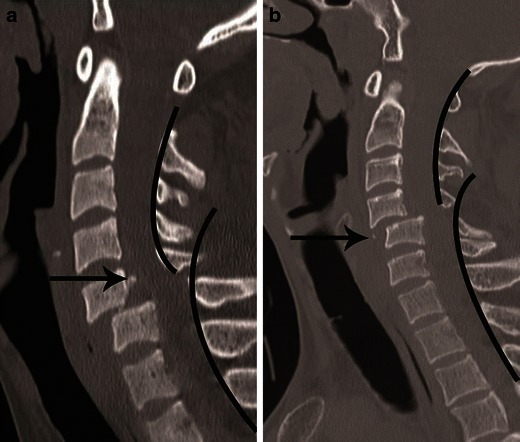
Bilateral versus unilateral facet joint dislocation. **a** Mid-sagittal CT image shows anterior translation of C5 over C6 by more than 50 % anteroposterior width of the C6, highly suggestive of bilateral facet joint dislocation, which was confirmed on parasagittal images (not shown). Large triangular bony fragment is noted at the posteroinferior corner of C5 (*arrow*) with diffuse loss of C5–C6 intervertebral disc space. **b** Mid sagittal CT image shows anterior translation of C4 over C5 by less than 50 % anteroposterior width of C5 (approximately 25 %), highly suggestive of unilateral facet joint dislocation, which was confirmed on parasagittal images (not shown). Small triangular bony fragment is noted at the anteroinferior corner of C4 (*arrow*) with loss of C4–C5 disc space. Disruption of spinolaminar line (*black curved lines*) at the level of anterolisthesis with anterior displacement of the same vertebra and lamina (C5 in **a** and C4 in **b**) is highly suggestive of hyperflexion injury. Presence of small avulsion fragments at the discovertebral junction with loss of intervertebral disc height is associated with disc injury, which needs further evaluation with MRI

**Fig. 15:**
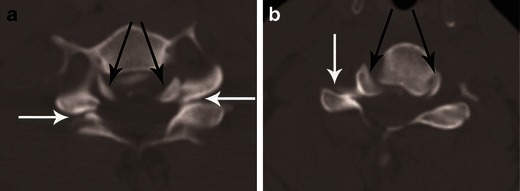
Bilateral versus unilateral facet joint dislocation. **a** Axial CT of the C5–C6 facet joint shows bilateral facet joint dislocation, suggested by reversal of the normal facet relationship, with convex surfaces opposing each other—called the “reverse hamburger sign” (*white arrows*). Loss of concentric relationship of the uncinate processes (*black arrows*) to the superior vertebral body—called the “positive headphone sign”. **b** Axial CT of the C4–C5 facet joint shows unilateral right-sided facet joint dislocation, suggested by a “naked facet” (*white arrow*)—due to absence of the opposing facet joint articular process because of dislocation. Loss of concentric arrangement of the right uncinate process (*black arrows*) due to rotation of vertebral body to the undislocated left side, resulting in unilateral positive “headphone sign”—suggesting rotational deformity

CT differentiation of unilateral versus bilateral facet joint dislocation is best appreciated in the sagittal plane (Fig. 14) [19, 30]. Though axial sections are not very useful to diagnose facet joint dislocation when isotropic sagittal reconstruction is possible, there are several well-described signs of diagnosing facet joint dislocation on axial images in the radiology literature, including the naked facet sign [31], the reverse “hamburger bun” [20] and the “headphones sign” (Fig. 15) [21]. A fracture of the posteroinferior cortex of the rotated vertebral body is frequently seen in patients with unilateral or bilateral facet joint dislocation. It is believed to be secondary to an avulsion of the PLL or the posterior annulus from the discovertebral junction [32].

Unilateral facet dislocation is mistakenly believed and reported as a stable injury in the literature; in reality it is a highly unstable injury that needs surgical stabilisation. Presence of an articular process in patients with facet joint dislocation is of surgical significance and should be reported. Unilateral facet joint dislocation was associated with fracture of articular processes in approximately 75 % of cases [32]. Presence of markedly comminuted fracture with displaced fracture might preclude a closed reduction of these injuries. Pre-operative MRI is recommended for the treatment decision in patients with bilateral and unilateral facet joint dislocation to look for intervertebral disc disruption. This information has huge therapeutic implication; because in the absence of disc disruption, these injuries are best treated by the posterior approach; in the event of disc damage, they need anterior decompression and stabilisation with fusion across the disc.

### Hyperextension injury

Hyperextension injury results when there is extreme extension of the spinal column, such as in a fall or vehicular collisions when the chin, forehead or face strikes an immovable object—like the dashboard, steering wheel or ground. Hyperextension injuries are observed in younger patients after high-energy trauma or in older patients with spondylotic or ankylosed spine after seemingly trivial injuries.

**Fig. 16:**
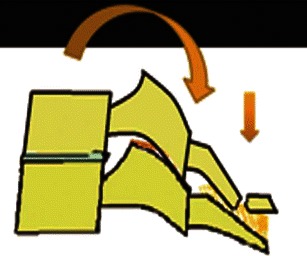
Schematic showing hyperextension compression injury. Extension vector with centre of rotation is behind the anterior column, resulting in axial loading force applied to the posterior column, resulting in compressive failure of the posterior column. With increasing magnitude of force, anterior column distraction and tensile failure might occur

Hyperextension injuries are biomechanically the opposite of hyperflexion injury, resulting in tensile failure of the anterior column and compressive failure of the posterior column. The Hyperextension injury spectrum includes both compressive hyperextension and distractive hyperextension, depending upon the fulcrum at the time of injury. Additional vectors like rotation and lateral flexion can result in complex hyperextension-rotation injury (Fig. 16).

#### Hyperextension compression

**Fig. 17:**
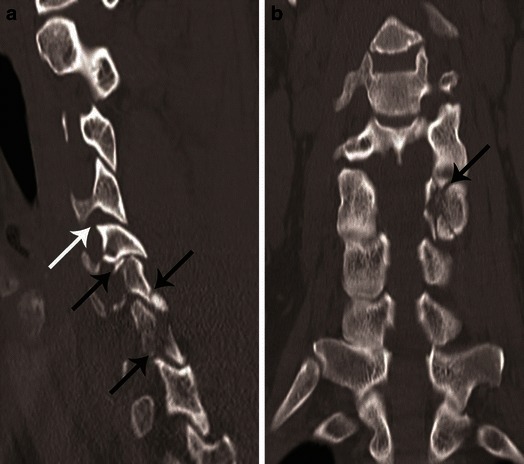
Hyperextension compression injury. **a** Parasagittal CT image shows C6, C7 articular pillar and process comminuted fracture with vertical orientation (*black arrows*). Focal anterior widening of C4–C5 facet joint (*white arrow*) is a characteristic feature of hyperextension injury. **b** Coronal CT image from the articular pillar in a different patient shows left C5 articular pillar fracture with vertical orientation and comminution (*black arrow*)

**Fig. 18:**
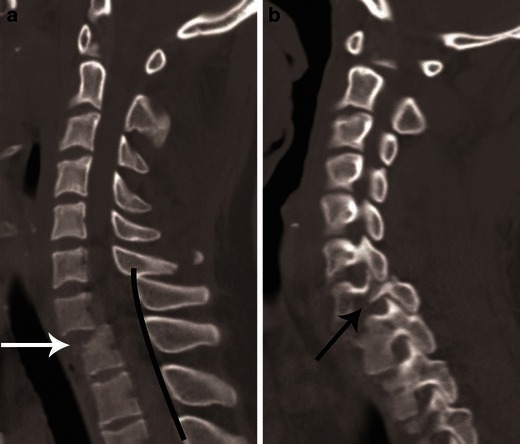
Hyperextension compression with traumatic spondylolisthesis. **a** Mid-sagittal CT image shows anterior listhesis of C7 over D1 (*white arrow*). This could result from hyperflexion distraction or, rarely, hyperextension injury. The spinolaminar line is continuous at the level of anterior translation, which suggests a hyperextension injury vector. **b** Left parasagittal image from the facet joint shows a vertically oriented fracture through the left pars interarticularis of C7 (*black arrow*) with impaction of the tip of the inferior articular process of C6 in the fracture gap—highly suggestive of hyperextension injury. Similar pars interarticularis fracture was also seen on the right side of C7 (not shown)

It represents axial load injuries in which the motion segment is extended and a compressive force is applied to the posterior motion segment, the neural arch (Fig. 16). The spectrum of injury in this group includes unilateral or bilateral fractures of the neural arch, including fractures of the lateral mass, laminae or articular pillars. Fractures can frequently be comminuted and occur at multiple contiguous levels (Fig. 17). Traumatic anterior spondylolisthesis occurs if there is bilateral pedicular or lateral mass fracture. This injury is commonly misdiagnosed as hyperflexion injury and spinolaminar line is very useful in differentiating between them (Fig. 18). The spinolaminar line is not interrupted at the level of anterolisthesis in hyperextension injury [23].

#### Hyperextension rotation

**Fig. 19:**
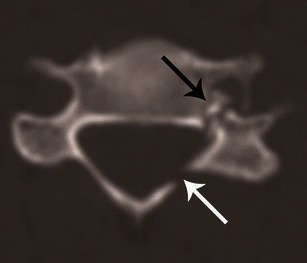
Pediculolaminar separation. Axial CT image shows simultaneous fracture of left lamina (*white arrow*) and pedicle (*black arrow*) of the C5 vertebra, resulting in pediculolaminar separation. Extension of the fracture line to the left transverse foramen and presence of fracture fragment within the transverse foramen seen, raising suspicion for the left vertebral artery injury

Typical injury results from eccentric extension force on the forehead or upper face with the head rotated, resulting in rotation and axial loading injury on the side of rotation. This causes an asymmetric, hyperextension compression injury of the posterior column, resulting in asymmetric or unilateral fractures of articular pillars or processes. Simultaneous fracture of the ipsilateral pedicle and lamina results in traumatic isolation of the cervical articular pillar (Fig. 19). The proposed injury mechanism is hyperextension rotation [33] and hyperflexion rotation [34]. Traumatic isolation of cervical articular pillar can potentially lead to rotational instability of the facet above and below the involved articular pillar and displaced fractures might need surgical stabilisation.

#### Hyperextension distraction

**Fig. 20:**
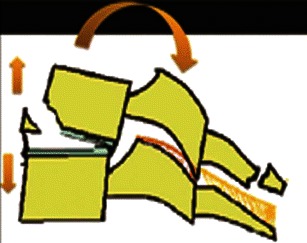
Schematic showing hyperextension distraction injury. Extension vector with centre of rotation posterior to the vertebral column, resulting in distraction and tensile failure of the anterior column. Increasing the magnitude of the force results in hyperextension dislocation

The centre of rotation of hyperextension distraction is posterior to the vertebral column with neck in extension, resulting in primary progressive tensile failure of the anterior column (Fig. 20). They are predominantly ligamentous disruptions that progress from anterior to posterior, beginning with the anterior longitudinal ligament. Tensile failure of the anterior column may be accompanied by compressive failure of the posterior column.

These injuries are very common in individuals with degenerated or ankylosed spines—as seen in ankylosing spondylitis and diffuse hypertrophic skeletal hyperosteosis (DISH)—even with relatively minor trauma [35].

**Fig. 21:**
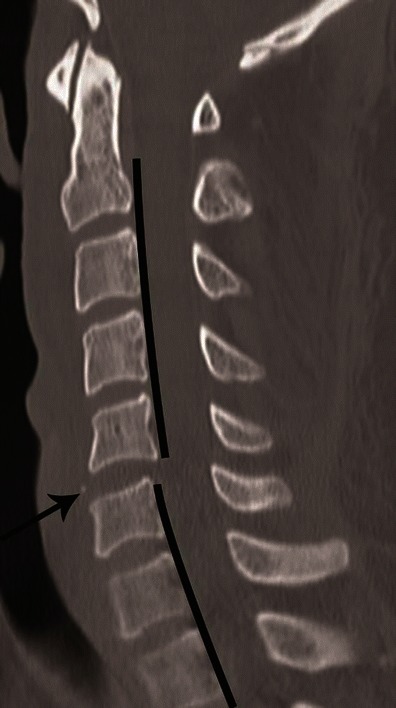
Hyperextension distraction with dislocation. Mid-sagittal CT image shows the significant prevertebral soft tissue swelling and subtle anterior widening of the C5–C6 disc space—a hallmark of hyperextension distraction injury. A small avulsion fracture (*arrow*) is noted at C5–C6. Mild retrolisthesis of C5 on C6 vertebral body suggested by disrupted posterior vertebral line (*black lines*)—suggestive of hyperextension dislocation

**Fig. 22:**
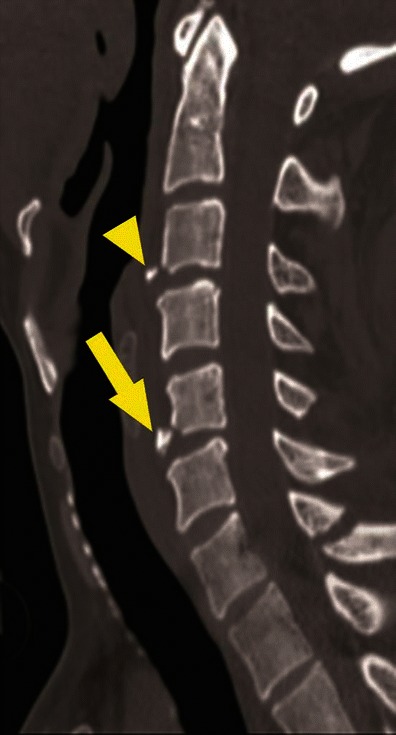
Hyperextension teardrop. Sagittal CT image shows small triangular avulsion fractures at the anteroinferior end plate of C3 (*arrowhead*) and C5 (*arrow*) without significant prevertebral soft tissue lesion. The triangular fragment at C5 is taller than wider, suggestive of hyperextension teardrop than hyperextension dislocation

CT in hyperextension distraction may show subtle abnormality, which can easily be overlooked due to the predominant soft tissue and ligamentous nature of the injury. Prevertebral soft tissue thickening can sometimes be the only clue to these injuries. Anterior disc space widening is the imaging hallmark of hyperextension distraction injury and results from ALL and anterior annulus rupture (Fig. 21) [33]. Tensile forces through the intact ALL may cause an avulsion fracture of the anterior body (an extension “teardrop” fracture) or fracture of an osteophyte or syndesmophyte overlying the disc space. CT scan shows extension teardrop fracture as a triangular fragment from the anteroinferior vertebral body or uncommonly from anterosuperior vertebral body on sagittal images (Fig. 22). In elderly patients with degenerated spines, C2 is most commonly involved, while in young patients the lower cervical spine is involved [33]. Facet joint injury resulting from hyperextension injury produces V-shaped facet joints that are wide anteriorly and tapered posteriorly (Fig. 17).

**Fig. 23:**
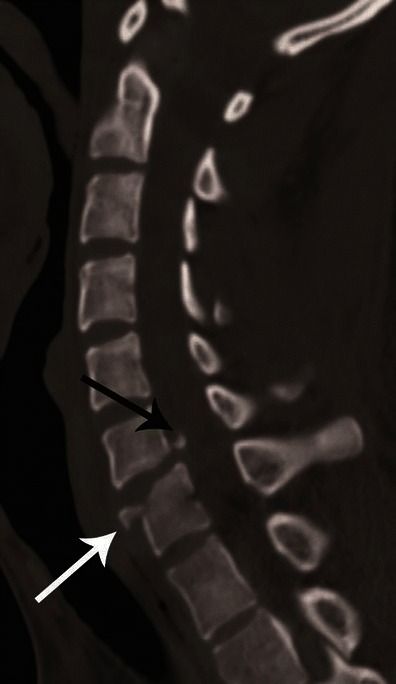
Avulsion fracture in hyperextension dislocation. Sagittal CT image shows an avulsion fracture at the anterosuperior end plate of C7 (*white arrow*), with the transverse diameter wider than the vertical diameter. Avulsion of the posteroinferior corner of the C6 vertebral body is also seen (*black arrow*), which suggests avulsion of the posterior annulus or PLL

Increasing hyperextension force results in hyperextension dislocation—transient posterior dislocation of the cranial vertebra over the caudal one—resulting in disruption of ALL, anterior annulus, disc, posterior annulus and PLL. The hyperextension dislocation injury is often accompanied by major neurological deficits, typically a central cord syndrome. Hyperextension-dislocation injuries sometimes produces minimal imaging features on radiographs or CT—as the posterior dislocation is transient and may be completely reduced spontaneously when the force is removed. It should be suspected in patients with lower facial trauma and central cord syndrome. The hallmark of distraction injury is widening of the anterior disc space and diffuse prevertebral soft tissue swelling. Some degree of retrolisthesis may also be present suggested by disrupted posterior vertebral line (Fig. 21). Less common CT indicators include: disc vacuum phenomenon and an avulsion fracture of the anteroinferior margin of the involved vertebra caused by avulsion due to the intact Sharpey’s fibres (Fig. 23) [23]. Horizontal fractures through the vertebral body or fused disc space might occur in ankylosed spines. MRI is indicated to further diagnose the extent of discoligamentous injuries and spinal cord compression/injury in patients with clinically or radiologically suspected hyperextension distraction injuries. MRI is complementary to CT in these cases and should be done in elderly or ankylosed spine with even trivial trauma and in the presence of any neurological deficit.

### Rotation injury

**Fig. 24:**
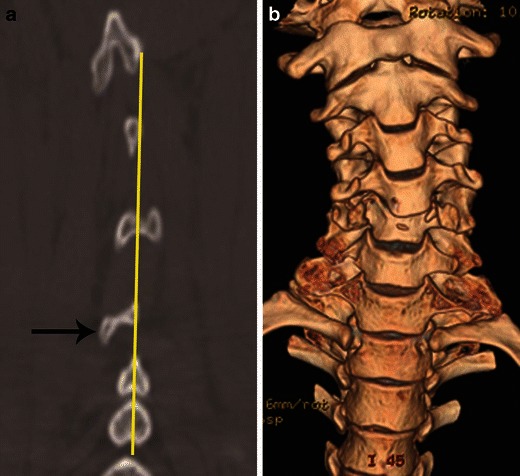
Rotational vector and deformity. **a** Posterior coronal CT image shows focal deviation of the C5 spinous process (*arrow*)—suggested by the interspinous line not passing through the rotated C5 spinous process. **b** Three-dimensional volume rendered coronal image shows rotational deformity of the C5/C6 vertebrae

Rotary injuries are the result of rotational or torsion force applied about the long axis of the vertebral column. Malalignment of spinous process on the coronal plane (Fig. 24a) is the most commonly used sign to imply the rotation vector; however, on CT, asymmetry of the uncinate process as seen on axial images is one of the most accurate and reliable indicator of the rotation vector (Fig. 15b) [21]. *Other useful signs suggestive of rotation injury include:* asymmetric disc space with rotated vertebral body in coronal plane and rotation of the fracture fragments. Rotational deformity is well depicted on 3D volume rendered image (Fig. 24b). Presence of rotation vector suggests more severe violence with possible ligamentous disruption.

#### Lateral flexion injury

Lateral flexion injuries are unusual, rarely isolated and most commonly occur in combination with hyperextension and are secondary to a rotational component at the time of impact. Asymmetrical compression of vertebral body suggests lateral flexion injury. Unilateral transverse process and uncinate process fracture typically results from lateral flexion injury. Although spinal cord injury is unusual, since the mechanism is thought to involve violent lateral flexion of the neck, nerve root injuries, including root avulsions and brachial plexus injuries, may occur.

## Injury morphology suggesting ligamentous injury

Although osseous injury to the cervical spine demands most of the attention, ligamentous integrity is equally important for the spine stability. Ligamentous disruption can occur in the absence of osseous abnormality, particularly with distraction injury vectors. Missed ligamentous injury can lead to significant morbidity and delayed instability, leading to persistent pain, kyphosis, delayed vertebral dislocation and neurological deficit.

Ligaments are not directly visualised on CT unlike on MRI. However, ligamentous failure can be inferred from CT by the presence of abnormal angulation, translation-dislocation and distraction injury morphology on CT (Table 3). Presence of normal CT is very reliable in excluding clinically significant unstable ligamentous injury [36]. MRI is traditionally used to diagnose suspected ligamentous injury with normal or equivocal CT.

**Table 3:** CT features predicting ligamentous and intervertebral disc injury

	Anterior column	Posterior column
Angulation rotation	Focal kyphosis	Focal displacement of spinous process on coronal plane
Translation/dislocation	Anterolisthesis Retrolisthesis	–
Distraction	Anterior disc space widening Posterior disc space widening	Interspinous widening Interlaminar widening
	Intervertebral disc injury: • Focal disc space widening • Avulsion at the anterior or posterior corner of vertebral body • Disc vacuum phenomenon and diffuse loss of disc space height	Facet joint distraction spectrum

## Look-alike injuries and usefulness of biomechanics in differentiation

Different biomechanical forces and injury vectors can produce “look-alike” injury morphology on CT. It is important to distinguish them, as these lesions may have different clinical implications.

The previous sections described the CT differentiation of unilateral versus bilateral facet joint dislocation and hyperextension distraction versus hyperextension dislocation injury. Important differentiating features between a burst versus flexion teardrop injury and flexion versus extension teardrop injury are explained in Tables 4 and 5 respectively.

**Table 4:** CT features differentiating flexion teardrop from burst fractures

Flexion teardrop (Fig. 8)	Burst fracture (Fig. 10)
Biomechanics—severe compressive flexion	Axial loading
Predominantly anterior VB compression—resulting in wedging	VB compression predominantly in the centre—resulting in diffuse loss of height
Oblique coronal fracture through the anteroinferior vertebral body. Fracture does not extend to the posterior cortex and posterior cortical line is intact (sagittal CT)	Fracture involves both anterior/posterior cortex with disruption of posterior cortical line (sagittal)
Comminution less severe—two/three fragments without centrifugal displacement (axial)	Comminution—severe with multiple fragments and centrifugal displacement
Retropulsion of the involved vertebral body along the rest of the suprajacent cervical spine Anterior fragment maintains alignment with the subjacent VB (sagittal/axial)	Retropulsion commonly from the postero-superior cortex (sagittal/axial) Rest of the spine above and below maintains alignment
Focal kyphosis	Normal curvature
Posterior column often distraction present—interspinous widening and facet distraction	Posterior column compression injury or normal
Mechanically unstable needs surgical stabilisation	Mechanically stable. Surgical treatment indicated for neural compromise from retropulsed bony fragments

**Table 5:** CT Features differentiating flexion and extension teardrop injuries

Flexion teardrop	Extension teardrop
Large fragment	Small triangular
Extends to both superior/inferior end plate	Involves only one end plate—anteroinferior or less commonly anterosuperior
Wedging/compression of VB	VB height is preserved
Retropulsion of fractured vertebral body	No retropulsion—Posterior vertebral line is intact
Focal kyphosis with flexion deformity	Normal curvature
Posterior column distraction present-interspinous widening and facet distraction	Posterior column compression injury or normal

*To predict vascular injury and the need for CT angiography*: The reported incidence of vertebral artery injury in patients who sustain cervical spine trauma is between 17 and 46 %. Most of them are asymptomatic [37–39]. Blunt vertebral artery injury is associated with complex cervical spine fractures involving subluxation, extension into the foramen transversarium or upper C1–C3 fractures [37–40]. CT angiography should be performed in patients with suspected vertebral artery injury.

## Biomechanics and management

Three critical surgical questions can be answered by biomechanics and injury vector based CT analysis:

1. Is there a mechanical instability that needs surgical stabilisation?
2. Which columns need stabilisation—anterior, posterior or circumferential?
3. Is there a need for short or long segment instrumentation?

An “unstable” cervical injury is commonly treated with operative stabilisation, while a “stable” injury may be managed conservatively by immobilisation by traction, collar or observation in bed. Fracture/ligamentous injury involving both load bearing (anterior) and tension (posterior) column suggest mechanical instability. Biomechanical vectors and injury morphology suggestive of distraction-rotation and shear-translation result in gross mechanical instability, which needs surgical stabilisation. Flexion teardrop injuries, bilateral facet dislocation and hyperextension distraction injury are also classified as mechanically unstable injuries and need surgical stabilisation. Isolated PLC injury in hyperflexion-distraction injury can results in delayed mechanical instability and should be stabilised and fused.

Anterior, posterior or circumferential stabilisation decision is based on the two essential principles in spine surgery, namely the “tension band principle” and the “load-sharing principle”. The column under tensile failure needs stabilisation first to reduce and align the displaced vertebral column; the vertebral body, if injured, can be reconstructed thereafter to share the load of body weight. Anterior longitudinal ligament disruption with disc injury, as occurs in hyperextension distraction injury, mandates anterior surgery with stabilisation and PLC injury as occurs in hyperflexion distraction, generally needs posterior reconstruction and stabilisation. Rotation-translation injuries may disrupt both anterior and posterior stabilising ligaments often requiring circumferential stabilisation.

The length of instrumentation is also often based on the force vectors involved. Significant translation and rotation vectors both require long segment stabilisation (two motion segments above and below injury).

## Summary

Cervical spine trauma CT can be interpreted utilising mechanistic principles as described in this article. This approach is based on the principle of identifying the “fingerprints” of the injury on MDCT, suggesting a particular injury mechanism, which leads to a systematic search for the entire spectrum of injuries based on the known pattern of injury produced by the particular biomechanics (Table 6). Both mechanistic and morphology based CT interpretation are complementary and the combination of mechanistic and morphology-based checklist—will help the radiologist to formulate surgically relevant and radiologically accurate report.

**Table 6:** CT fingerprints and injury vectors

Biomechanics	Anterior fingerprints	Posterior fingerprints
Hyperflexion	Anterior column compression	Posterior column distraction
	Curvature: focal kyphosis Alignment: anterolisthesis VB: wedge compression and flexion teardrop. Burst/coronal split: axial loading Disc space: focal posterior widening or diffuse narrowing.	Facet joint: diffuse widening more than 2 mm, focal posterior widening, subluxation and dislocation Interspinous widening
Hyperextension	Anterior column distraction	Posterior column compression
	Curvature: hyperlordosis or normal Alignment: normal or retrolisthesis VB: extension teardrop Disc space: focal anterior widening/normal	Articular pillar/process fracture Lamina/spinous process fracture Pediculo-laminar separation
Lateral flexion	Always coupled with rotation. Frequently associated with hyperextension and hyperflexion. Reciprocal compressive and distractive injury on right/left side Curvature: coronal plane tilt VB: lateral compression injury on the side of flexion Asymmetrical loss of disc height in coronal plane	Reciprocal compressive and distractive injury on right/left side Unilateral articular pillar or laminar fracture. Facet joint distraction on the side opposite of posterior element fracture
Rotation	Usually associated with flexion, extension	Unilateral facet dislocation or fracture Asymmetric posterior column injury

*VB* vertebral body
